# CHCHD2 is a potential prognostic factor for NSCLC and is associated with HIF-1a expression

**DOI:** 10.1186/s12890-020-1079-0

**Published:** 2020-02-13

**Authors:** Xin Yin, Jinghua Xia, Ying Sun, Zhipei Zhang

**Affiliations:** 1 Department of Radiotherapy, Xianyang Hospital, Yanan University, Xianyang, Shaanxi 712000 People’s Republic of China; 2 Department of Thoracic Surgery, The Second Affliated Hospital of Air Force Medical University, Xi’an, 710038 China

**Keywords:** CHCHD2, HIF-1α, Non-small cell lung cancer, Prognostic

## Abstract

**Background:**

CHCHD2 was identified a novel cell migration-promoting gene, which could promote cell migration and altered cell adhesion when ectopically overexpressed in NIH3T3 fibroblasts, and it was identified as a protein necessary for OxPhos function as well. However, the clinic relevance of CHCHD2 expression in NSCLC remains unclear. Here we assumed that CHCHD2 expression would accompanies the expression of HIF-1α to response hypoxia in the occurrence of NSCLC.

**Methods:**

In order to verify this hypothesis, correlations among the expression levels of CHCHD2 and HIF-1α were detected and analyzed in 209 pair cases of NSCLC. The expression and location of these molecules were assessed using Immunohistochemistry, immunohistofluorescence, qRT-PCR and western blotting. The differences and correlations of the expression of these two molecules with clinical pathological characteristics in NSCLC were statistically analyzed using Wilcoxon (W) text, Mann-Whitney U, Kruskal-Wallis H and cross-table tests. Kaplan-Meier survival analysis and Cox proportional hazards models were used to estimate the effect of the expression of CHCHD2 and HIF-1α on the patients’ survival.

**Results:**

Data showed that CHCHD2 and HIF-1α expression were higher in NSCLC than in normal tissues (all *P* = 0.000). CHCHD2 expression was significantly related with smoking, tumor size, differentiation degree, TNM Stage, lymph metastasis (all *P<*0.05). The HIF-1α expression was significantly associated with smoking, tumor category, differentiation degree, TNM Stage, Lymph metastasis (all *P<*0.05). There was a marked correlation of CHCHD2 and HIF-1α expression with histological type, differentiation and lymph metastasis of NSCLC (all *P<*0.05, r_s_*>*0.3). Immunohistofluorescence showed that there were co-localization phenomenon in cytoplasm and nucleus between CHCHD2 and HIF-1α expression. NSCLC patients with higher CHCHD2 and HIF-1α expression had a significantly worse prognosis than those with lower CHCHD2 and HIF-1α expression (all *P =* 0.0001; log-rank test). The multivariate analysis indicated that CHCHD2 expression was an independent prognostic factor in NSCLC (hazard ratio [HR], 0.492, *P =* 0.001).

**Conclusion:**

Our results indicate that over-expression of CHCHD2 would promote the expression of HIF-1α to adapt the hypoxia microenviroment in NSCLC and CHCHD2 could serves as a prognostic biomarker in NSCLC.

## Background

Lung cancer is the leading cause of cancer-related mortality in both men and women worldwide [1]. More than 80% of lung cancer are non-small-cell lung cancer (NSCLC), which is comprised mostly of squamous cancers and adenocarcinomas [2, 3]. According to survey of the American Cancer Society (ACS), lung cancer accounts for 28% of all male cancer deaths and 26% of all female cancer deaths in 2014 [4]. As lung cancer cells infiltrate into surrounding tissue and metastasize to distant organs, the 5-year survival rate in lung cancer patients was very low. In spite of improvements in surgical, radiotherapy, platinum-based doublet chemotherapy and recently developed targeted therapies [5], the prognosis of NSCLC is still very poor and about 30–55% of the patients who are diagnosed early and treated by surgery will develop a recurrence [6, 7]. The targeted therapies interrupt signaling pathways which responsible for lung cancer cell proliferation and survival [5]. Hence, it is urgent to understand the potential molecular mechanisms mediating the NSCLC tumorigenesis and identify novel biomarkers to help provide individualization treatment and assess better prognosis [8, 9].

According to the NCBI database (http://www.ncbi.nlm.nih.gov/), Coiled-coil-helix-coiled-coil-helix domain-containing protein 2 (CHCHD2) also named Mitochondria Nuclear Retrograde Regulator 1 (MNRR1) was located on human chromosome 7p11.2, containing 4 exons, 456 nucleotide bases and encoding 151 amino acids. It was identified as a member of the protein family which containing a coiled-coil-helix-coiled-coil-helix (CHCH) domain. There was a single putative that CHCHD2 consists of an N-terminal mitochondrion localization sequence and a strongly conserved C-terminal CHCH domain, and they were together performed their function in the cells [10]. CHCHD2 was a gene that has not yet been clearly defined, and previous research revealed that CHCHD2 played a role in translation in human cells [11]. Furthermore the CHCHD2 cDNA was found to promote cell migration and altered cell adhesion when ectopic overexpressed in NIH3T3 fibroblasts [10]. Subsequently, CHCHD2 was found highly expressed in the HCC specimens [12] and co-amplified with EGFR in NSCLC [13], however, the expression of CHCHD2 in NSCLC and how will that affect the progress and prognosis of the NSCLC was not confirmed. To study the clinicopathologic features and prognostic implications of CHCHD2 expression in patients with NSCLC, we investigated the expressions of CHCHD2 in NSCLC by immunohistochemical staining, Real Time quantitative PCR and Western blot, and assessed the relationships between CHCHD2 and clinical parameters.

In computational screen, CHCHD2 was identified as a protein necessary for OxPhos function [14]. Further research demonstrated that under reduced oxygen tension microenvironment, CHCHD2 expression was enhanced [15]. Hypoxia was typically associated with many types of solid tumors [16], Hypoxia-inducible factor-1 (HIF-1) was a principle modulator of the tumor cell response to hypoxia, and HIF-1α level was normally kept low by proteasomal degradation, it rapidly stabilized under conditions of hypoxia [17]. Given all these elements, we speculated that CHCHD2 might was a novel molecular to regulator HIF-1α function and hypoxia signaling. Thus, the present study also focused on the correlation of CHCHD2 and HIF-1α expression in NSCLC, suggesting that CHCHD2 might interacted with HIF-1α to response hypoxia in the occurrence of NSCLC, in order to provide a basis for further study on the developmental mechanism of CHCHD2 in regards to NSCLC.

## Methods

### Patients and tissue samples

A total of 209 NSCLC patients tissue specimens were collected from 2006 to 2011, admitted by Department of Thoracic Surgery of Tangdu Hospital. All of these enrolled patients did not receive chemotherapy or targeted therapy. The detailed clinicopathologic features information of the patients were obtained from the medical records in a computerized registry database were summarized in Table 2. The patients median age was 62 years (range, 31–81 years). The operation date is considered as the starting date for estimating the postoperative survival time, and the deadline for follow-up was 30 July, 2016. The median follow-up period of these patients was 36 months (range, 0–79 months). The research protocol was approved by the Regional Ethics Committee for Clinical Research of the Air Force Medical University. All patients provided written informed consent and agree to use their medical records and tissue samples for research purposes.

### Immunohistochemistry

The expression of CHCHD2 and HIF-1α protein in all the tissue sections of the subjects were investigated by rabbit antibodies (anti-CHCHD2, diluted 1:50, ABGENT, SanDiego, CA or anti-HIF-1α, diluted 1:100, Proteintech™, Chicago, USA). The detailed operation process and staining score of immunohistochemistry accordance with our previous methods [18]. The final immunohistochemical staining score reported is the average of the scores from the tow investigators.

### RNA isolation and quantitative RT- PCR

Extraction of total RNA from frozen tissues was performed using TRIzol reagent (Invitrogen, USA). RevertAid First-Strand cDNA Synthesis Kit (Thermoscientific, Vilnius, Lithuania) was used to prepare reverse transcription according to the manufacturer’s protocols. The *qRT-PCR* reaction was carried out using LightCycler-Fast-Start DNA Master SYBR Green (Roche Diagnostics, Tokyo, Japan). Gene expression was normalized to β-actin. Three parallel reactions were set for each sample. The mRNA expression value were calculated by the MxPro QPCR Software 4.10 (Mx3005P, Stratagene, Agilent Technologies) according to 2-^△△CT^ method. The primers used for *qRT-PCR* were: CHCHD2, F-5′- CAG TTG GCT CTTBCTGBCTG CT-3′ and R-5′-GTA ATG GCG TGA CCC AAT GT-3′; HIF-1α, F-5′-TGC AAC ATG GAA GGT ATT GC − 3′ and R-5′-TTC ACA AAT CAG CAC CAA GC − 3′; β-actin, F-5′-TCC CTG GAG AAG AGC TAC GA-3′ and R-5′-AGC ACT GTG TTG GCG TAC AG-3′.

### Western blot

The frozen Tissue was cut into pieces and placed in RIPA Lysis Buffer (P0013B, Beyotime Biotechnology) containing protease inhibitor (Phenylmethanesulfonyl fluoride, PMSF, ST506, Beyotime Biotechnology) on ice for 1 h. Then the mixture was homogenized, centrifuged at 12,000 rpm for 20 min at 4 °C. The supernatant was collected and protein concentration was determined using the Pierce BCA Protein Assay Kit (Thermo Fisher Scientific). Total proteins were separated by electrophoresis using SDS-PAGE gel and transferred onto PVDF membrane. After by blocking in 9% not-fat milk, the membrane was incubated with specific antibodies overnight at 4 °C (anti-CHCHD2, 1: 500; anti-HIF-1α 1:300; anti-β-actin, 1: 1000). Then washing with TBST for 30 min, the membrane was incubated with a Rabbit anti-Human secondary antibody (1:5000) for 1 h at temperature. For each membrane, band intensity was analyzed using the Millipore chromogenic kit (Millipore, Billerica, MA, USA) and quantitatively analyzed using Quantity software (Bio-Rad, USA).

### Immunohistofluorescence

According to immunohistochemistry procedures, the slides were incubated with primary antibodies (anti-CHCHD2, 1:50) and stained with goat anti-rabbit (Alexa Fluor 488; Zhuangzhi Bio, Xi′an, China), washed with PBS. Then the slides were incubated with anti-HIF-1α (diluted 1:100) antibodies and stained with goat anti-rabbitat (Cy3; Zhuangzhi Bio, Xi′an, China). After the final washing, the slides were mounted in 50% glycerol (in PBS) and examined by a fluorescence microscope (Leica DM4000B, Leica, Wetzlar, Germany).

### Oncomine analysis

In order to further explain the expression level of CHCHD2 in NSCLC and its prognostic value, we used Oncomine database (https://www.oncomine.org/) to analyze. Search the target gene CHCHD2, and filter as follows, Analysis Type: Lung Cancer vs. Normal Analysis, Sample Type: Surgical Specimen. Select the reporter (217720_at) for meta-analysis. The sample names, tissue types, and expression values (log2 median-centered intensity) of the included data sets were recorded, and the mRNA expression level of CHCHD2 was statistically analyzed using Graphpad Prism 5 software. Search the target gene CHCHD2, and filter as follows, Cancer Type: Lung Cancer, Clinical Outcome: Survival Status, Sample Type: Surgical Specimen. Record the Sample name, Tissue type and Expression value, Survival time, Survival status. The median of gene expression value as cut off value, and gene expression was divided into low and high expression. Survival analysis with Graphpad prism 5.

### Statistical analysis

Statistical analyses were done with SPSS17.0 Software (SPSS Inc., Chicago, IL, USA). The Wilcoxon (W) text was used to evaluate the comparison of CHCHD2 and HIF-1α protein expression between NSCLC and corresponding normal tissue. Associations between immunohistochemical expression and clinical variables were evaluated by Mann-Whitney *U* test (among tow groups), Kruskal-Wallis *H* test (among multiple groups) and Spearman’s rank correlation analysis as appropriate. *r*_*s*_*>*0.3, *P<*0.05 was considered to be statistically significant. The Cox proportional hazards model was used for univariate and multivariate analyses. Survival curves were examined using the Kaplan-Meier method, and compared using the log-rank test of GraphPad Prism software version 5 (GraphPad Software, Inc., CA, USA).

## Results

### CHCHD2 and HIF-1α overexpressed in NSCLC tissue

**Fig. 1:**
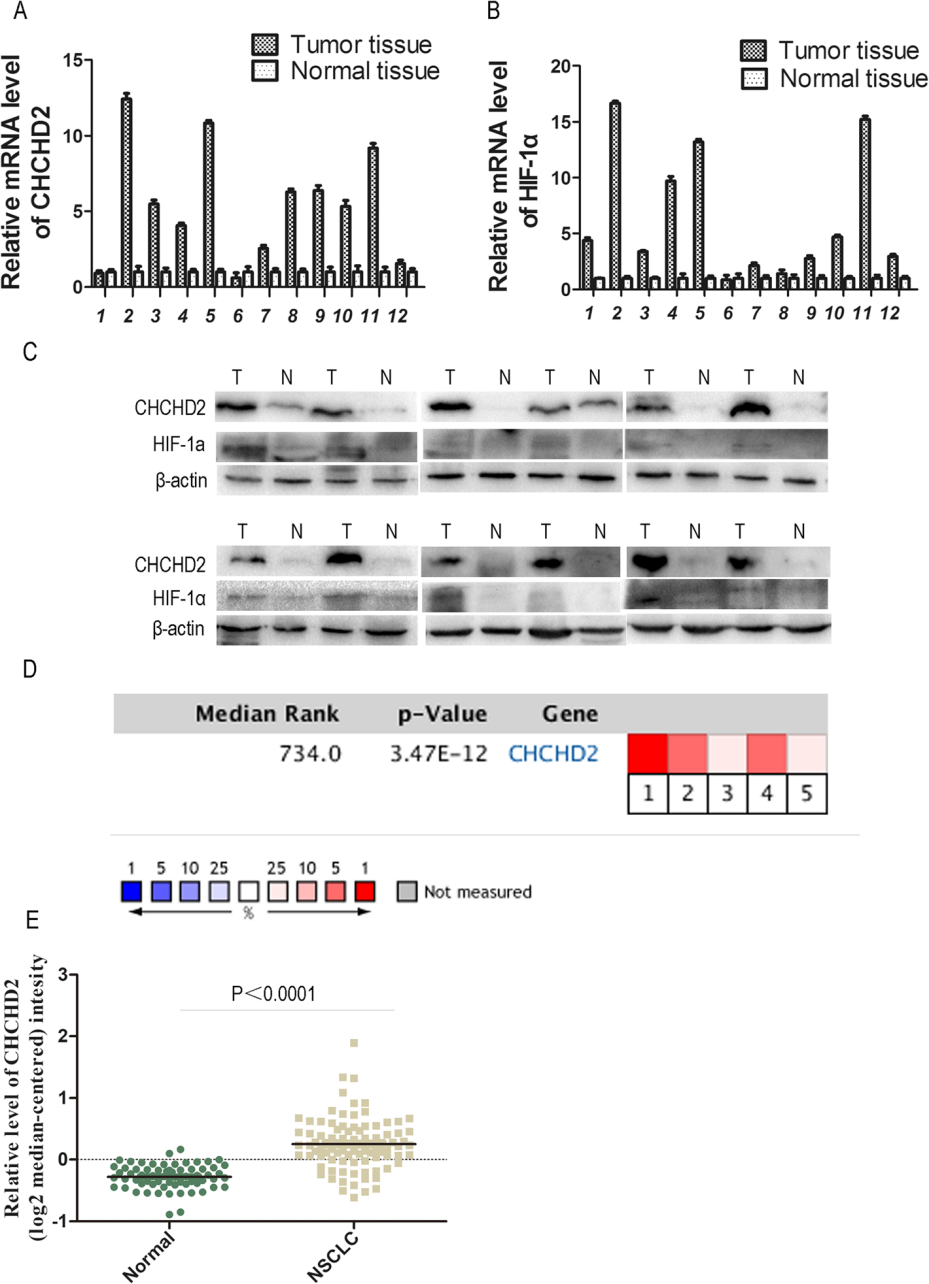
CHCHD2 and HIF-1α overexpressed in NSCLC tissue. **a** Expression of CHCHD2 mRNA was higher in tumor tissue than that in paired normal tissue. **b** Expression of HIF-1α mRNA was higher in tumor tissue than that in normal tissue. **c** Expression of CHCHD2 and HIF-1α protein were higher in tumor tissues (T) than those in pair normal tissues (N), and β-actin was used as an internal control. **d** A meta-analysis of CHCHD2 mRNA expression from Oncomine databases where colored squares indicate comparative analysis of CHCHD2 expression in specific data subsets. (1) LCC vs. Normal, Hou Lung [19], (2) ADC vs. Normal, Hou Lung [19], (3) SCC vs. Normal, Hou Lung [19], (4) ADC vs. Normal, Okayama Lung [20], (5) ADC vs. Normal, Su Lung [21], **e** Expression of CHCHD2 mRNA was higher in tumor tissue than that in normal tissue

We collected 12 paired fresh tumor and normal tissue samples, CHCHD2 and HIF-1a mRNA and protein expression levels in those tissues were detected by qRT-PCR and Western blot respectively. The results showed that the mRNA and protein expression levels of CHCHD2 and HIF-1a in tumor tissues were significantly higher than those in the normal tissues (Fig. 1a-c).

According to the datas Hou Lung [19], Okayama Lung [20], Su Lung [21] (5 types data) contained in the oncomine database, a meta-analysis was carried out. Compared with normal tissues, CHCHD2 was highly expressed in NSCLC with a median rank value of 734.0 (*P* = 3.47e-12) (Fig. 1d). Based on the included data, the mRNA expression level of CHCHD2 in NSCLC was statistically higher than normal tissues, *P* < 0.0001 (Fig. 1e).

**Fig. 2:**
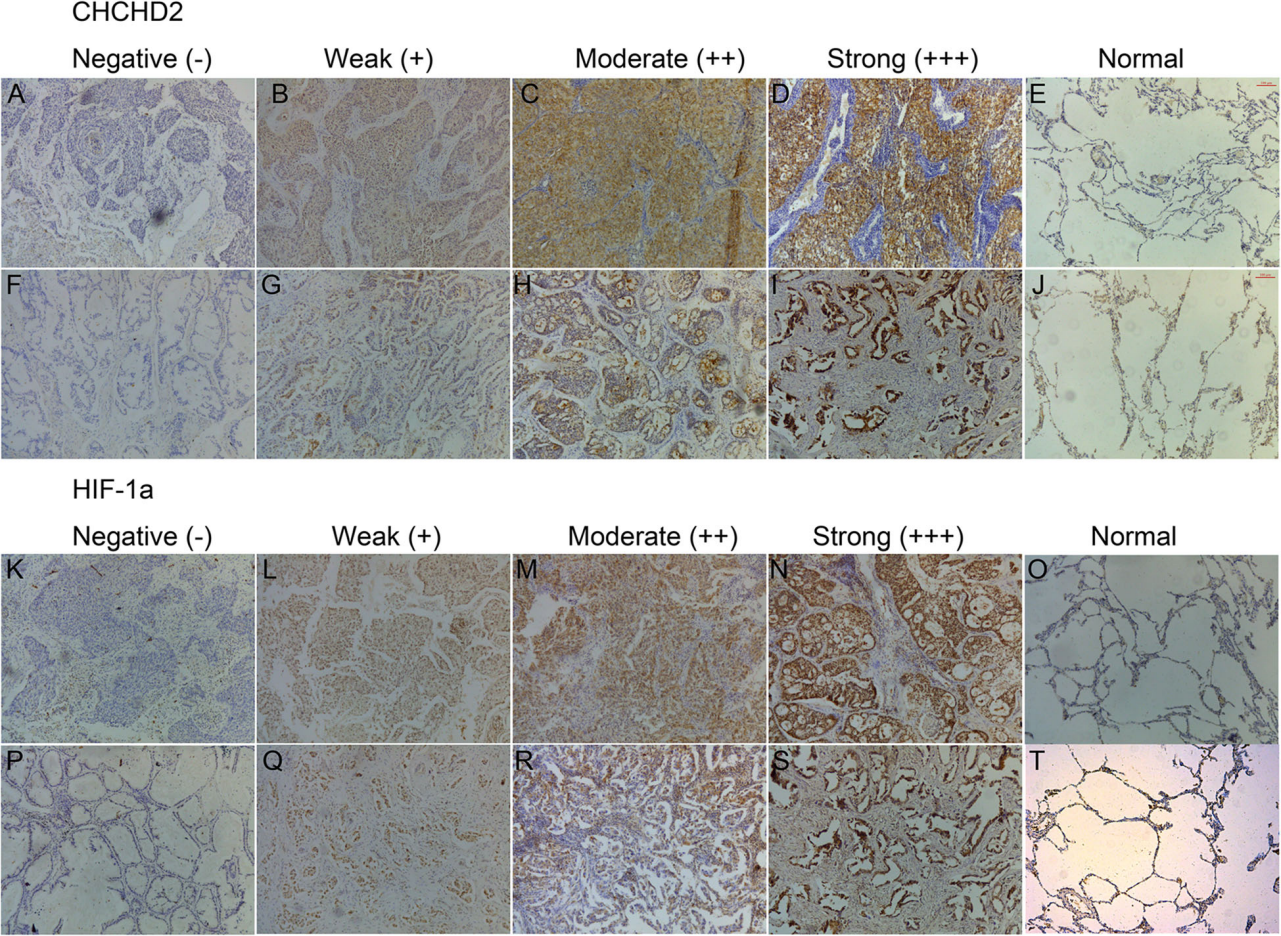
Representative immunohistochemistry staining of CHCHD2 and HIF-1αin NSCLC and normal tissue samples. **a-j** were CHCHD2 staining in NSCLC and normal tissues. **a-d** were squamous cell carcinoma, **f-i** were adenocarcinoma, **e, j** were normal tissues, **a, f** were negative staining, **b, g** were weak positive staining, **c, h** were moderate positive staining, **d, i** were strong positive staining; **e** was negative staining in normal tissue, and **j** was positive staining in normal tissue. Figure **k**-**t** were HIF-1α staining in NSCLC and normal tissues. **k-n** were squamous cell carcinoma, **p-s** were adenocarcinoma, **o, t** were normal tissues, **k, p** were negative staining, **l, q** were weak positive staining, **m, r** were moderate positive staining, **n, s** were strong positive staining; **o** was negative staining in normal tissue, **t** was positive staining in normal tissue. (All images are magnified at × 100)

In addition, we detected the protein expressions of CHCHD2 and HIF-1a in tumor tissues and adjacent non-cancerous tissues by immunohistochemistry in specimens from 209 patients. The results showed that the positive stain of CHCHD2 and HIF-1a were 94.7% (198/209) and 95.7% (200/209) in tumor tissues, there were significantly higher than that in adjacent non-cancerous tissues 11.0% (23/209) and 68.9% (144/209), (*P* = 0.000, P = 0.000) respectively (Table 1). Positive staining of CHCHD2 and HIF-1α were all located in the cytoplasm and nucleus (Fig. 2).

**Table 1:** The difference expression levels of CHCHD2 and HIF-1α protein in NSCLC and normal tissue

	Normal	CHCHD2						Normal	HIF-1α					
		–	+	++	+++	total	Wilcoxon (*W*) text		–	+	++	+++	total	Wilcoxon (*W*) text
NSCLC	–	11	79	85	11	186	*Z =* -12.439 *P =* 0.000	–	8	20	32	5	65	*Z =* -19.244 *P =* 0.000
	+	0	3	16	4	23		+	1	22	83	9	115	
	++	0	0	0	0	0		++	0	6	15	8	29	
	total	11	82	101	15	209		total	9	48	130	22	209

### Association between expressions of CHCHD2, HIF-1α and clinicopathologic features

In order to study the clinical significance of CHCHD2 and HIF-1a expression in NSCLC, we analyzed the relationship between CHCHD2 and HIF-1a and clinicopathological characteristics of NSCLC (Table 2). The result indicates that the CHCHD2 expression was significantly associated with smoking (*P* = 0.045), tumor size (*P* = 0.000), differentiation degree (*P* = 0.034), TNM stage (*P* = 0.000), lymph metastasis (*P* = 0.000), but there was no significant of CHCHD2 expression correlation with gender (*P* = 0.139), age (*P* = 0.567), tumor position (*P* = 0.306) and tumor category (*P* = 0.082). With a similar manner, we found the HIF-1α expression was significantly associated with smoking (*P* = 0.035), tumor category (*P* = 0.027), differentiation degree (*P* = 0.003), TNM Stage (*P* = 0.002), lymph metastasis (*P* = 0.003), however there was no significant of HIF-1α expression correlation with gender (*P* = 0.170), age (*P* = 0. 549), tumor position (*P =* 0.871) and tumor size (cm) (*P =* 0.489).

**Table 2:** Association between CHCHD2 and HIF-1α protein expressions and clinicopathological features in NSCLC patients

Clinicopathologic features	*n* = 209	CHCHD2 expression						HIF-1α expression					
		–	+	++	+++	*Z*	*P*-value	–	+	++	+++	*Z*	*P*-value
Gender													
Male	157	11	63	70	12	−1.478	0.139	8	39	94	16	−1.371	0.170
Female	52	0	18	29	5			1	9	36	6		
Age (years)													
≥62	107	7	37	56	7	−0.573	0.567	5	23	66	13	−0.599	0.549
< 62	102	4	45	45	8			4	25	64	9		
Smoking													
Yes	132	10	54	61	7	−2.001	**0.045**	8	36	74	14	−2.114	**0.035**
No	77	1	28	40	8			1	12	56	8		
Tumor position													
Periphery type	73	3	34	31	5	−1.024	0.306	4	14	50	5	−0.162	0.871
Central type	136	8	48	70	10			5	34	80	17		
Tumor size (cm)													
>5	109	5	27	69	8	−3.916	**0.000**	5	23	68	13	−0.692	0.489
≤5	100	6	55	32	7			4	25	62	9		
Tumor category													
Adenocarcinoma	82	2	29	44	7	−1.738	0.082	2	12	59	9	−2.213	**0.027**
Squamous caner	127	9	53	57	8			7	36	71	13		
Differentiation degree													
Well/ Moderate	152	8	66	70	8	−2.122	**0.034**	7	42	91	12	−2.939	**0.003**
Poor	57	3	16	31	7			2	6	39	10		
TNM Stage													
Ia-IIa	116	9	68	38	1	−7.279	**0.000**	8	33	66	9	−3.093	**0.002**
IIb -IIIb	93	2	14	63	14			1	15	64	13		
Lymph metastasis													
metastasis	116	4	33	65	14	−4.468	**0.000**	3	20	77	16	−2.967	**0.003**
Non-metastasis	93	7	49	36	1			6	28	53	6		

### Relationship between clinicopathological features and survival in NSCLC patients

**Fig. 3:**
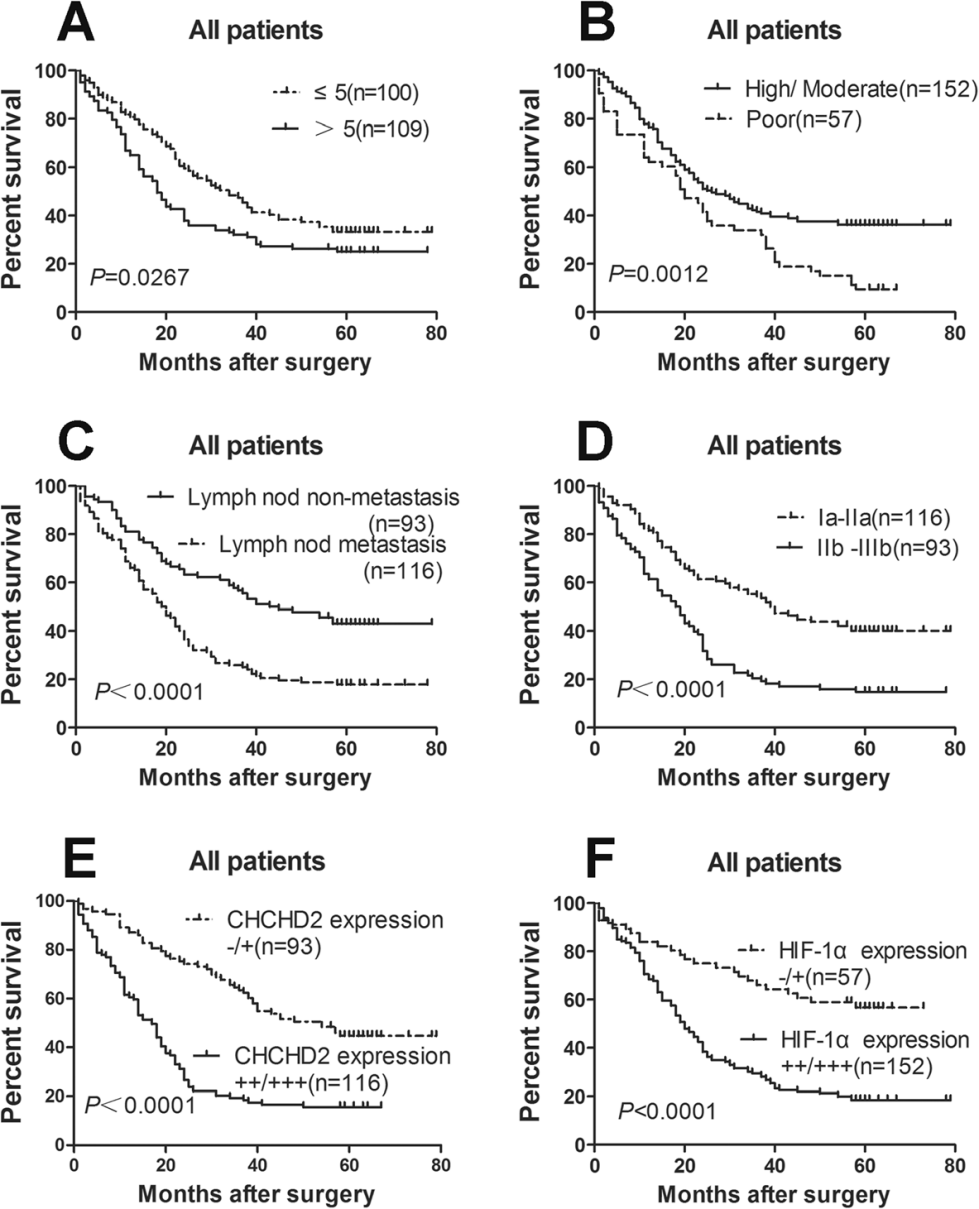
Kaplan-Meier survival analysis of the NSCLC patients. The *P*-value was determined using the log-rank test. **a** Comparison of the overall survival (OS) between tumor size ≥5 cm and < 5 cm NSCLC patients. **b** Comparison of the OS of patients between well/moderately differentiated and poorly differentiated NSCLC tissues. **c** Comparison of OS between TNM Ia-IIa and IIb -IIIb NSCLC patients. **d** Comparison of the OS between lymph node metastasis and non-lymph node metastasis NSCLC patients. **e** Comparison of the OS between low CHCHD2 expression (−/+) and high CHCHD2 expression (++/+++) NSCLC patients. **f** Comparison of the OS between low HIF-1αexpression (−/+) and high HIF-1αexpression (++/+++) NSCLC patients

Among factors in Table 2, the tumor size, TNM stage, differentiation and lymph node metastasis were significantly associated with the expression of CHCHD2, here we analyzed the relationship between these factors and patient’s survival. The median survival time of 100 patients with tumor size ≤5 cm was 32 months (95% confidence interval [CI] = 22.2–41.8 months), while that of 109 patients with tumor size > 5 cm was 18 Months (95% CI = 14.1–21.9 months). Tumor size will affect the survival time of patients (*P* = 0.0267) (Fig. 3a). One hundred fifty two patients with well or moderately differentiated tumors (median survival time was 24 months, 95% CI = 17.3–30.7 months) had a longer survival time than 57 patients with poorly differentiated tumors (median survival was 19 months, 95% CI = 13.7–24.3) (*P* = 0.0012) (Fig. 3b). The median survival time of 93 patients with no lymph node metastasis was 40 months (95% CI = 23.7–56.3 months), whereas the 116 patients with lymph node metastasis tumors has shorter median survival time was 18 months (95% CI = 13.6–22.4 months) (*P* < 0.0001) (Fig. 3c). The median survival time of 116 patients with TNM Ia-IIa tumors was 39 months (95% CI = 29.2–48.8 months), whereas the 93 patients with TNM IIb-IIIb has shorter median survival time was 17 months (95% CI = 11.9–22.1 months) (P < 0.0001) (Fig. 3d). These results show that patients with tumor size > 5 cm, poorly differentiation, high TNM stage and lymph node metastasis have a shorter survival time.

### High levels of CHCHD2, HIF-1α predict poor prognosis of NSCLC patients

In order to explore whether the expression of CHCHD2 and HIF-1a are prognostic factors for NSCLC, we investigate the correlation between the expressions of CHCHD2 and HIF-1a and patient survival. Patients who expressed CHCHD2 and HIF-1a as −/+ were considered as poor expression, while patients who expressed as ++/+++ were considered as high expression. The median survival time of patients (*n* = 116) with high expression of CHCHD2 was 15 months (95% CI = 11.9–18.1 months), and the median survival time of patients (*n* = 152) with high expression of HIF-1α was 19 months (95% CI = 16–22 months), whereas the median survival time of patients with poor expression of CHCHD2 and HIF-1a had not yet been reached. The mean survival time of patients with high expression of CHCHD2 and HIF-1a was 21.6 months (n = 116, 95% CI = 17.7–25.4 months) and 28.6 months (n = 152, 95% CI = 24.4–32.8 months), respectively. Patients with poor expression of CHCHD2 and HIF-1a had a longer mean survival time of 50.8 months (*n* = 93, 95% CI = 45–56.5 months) and 50.2 months (*n* = 57, 95% CI = 42.8–57.5 months). These results show that patients with high expression of CHCHD2 and HIF-1a had a shorter survival time and patients with high expression of CHCHD2 and HIF-1a had a significantly worse prognosis than those with poor expression of CHCHD2 and HIF-1a (*P* < 0.0001) (Fig. 3e-f).

To further clarify whether CHCHD2 expression is a prognostic factor in patients with NSCLC, a Cox’s proportional hazards model was used for regression analysis. We first performed a univariate analysis of the clinicopathological characteristics of NSCLC contained CHCHD2 and HIF-1a expression, according to the result of the univariate analysis, factors contained CHCHD2 (*P* = 0.000) and HIF-1α (P = 0.000) expression, tumor size (*P* = 0.012), grade of differentiation (*P* = 0.001), TNM stage (P = 0.000) and lymph node metastasis (P = 0.000) showed significantly higher hazard rations for a poor prognosis (Table 3). Based on this, multivariate analysis was carried out using the significant factors observed in univariate analysis. The results showed that, the grade of differentiation (*P* = 0.026), lymph node (*P* = 0.014), CHCHD2 (P = 0.001) and HIF-1α (*P* = 0.015) expression were all an independent prognostic factor respectively. These results strongly indicated that the CHCHD2 expression and HIF-1α expression in NSCLC were closely related to patient’s poor prognosis (Table 3).

**Table 3:** Cox proportional hazards model analysis of variables affecting survival in NSCLC patients

Variables	Categories	Univariate analysis		Multivariate analysis	
		HR(95% CI)	*P*-value	HR(95% CI)	*P*-value
Sex	Male/Female	1.268 (0.885–1.816)	0.196		
Age (years)	<61/≥61	1.012 (0.734–1.395)	0.940		
Smoking history	Smoking/Non-smoking	1.200 (0.864–1.665)	0.277		
Tumor position	Periphery type/Central type	0.937 (0.670–1.309)	0.702		
Tumor size (cm)	≤5/>5	1.511 (1.094–2.087)	**0.012**	0.808 (0.561–1.164)	0.252
Histological type	Adenocarcinoma/Squamous	0.931 (0.671–1.291)	0.669		
Grade of differentiation	Well+moderate/poor	1.814 (1.294–2.542)	**0.001**	0.668 (0.468–0.952)	**0.026**
TNM Stage	Ia~IIa/IIb~IIIb	2.204 (1.592–3.052)	**0.000**	1.076 (0.674–1.781)	0.760
Lymph metastasis	Metastasis/Non-metastasis	0.491 (0.351–0.688)	**0.000**	1.661 (1.110–2.486)	**0.014**
CHCHD2 expression	-~+/++~+++	2.966 (2.100–4.188)	**0.000**	0.492 (0.321–0.752)	**0.001**
HIF-1α expression	-~+/++~+++	2.866 (1.859–4.421)	**0.000**	0.549 (0.338–0.891)	**0.015**

### The co-localization of CHCHD2 with HIF-1α protein expression

**Fig. 4:**
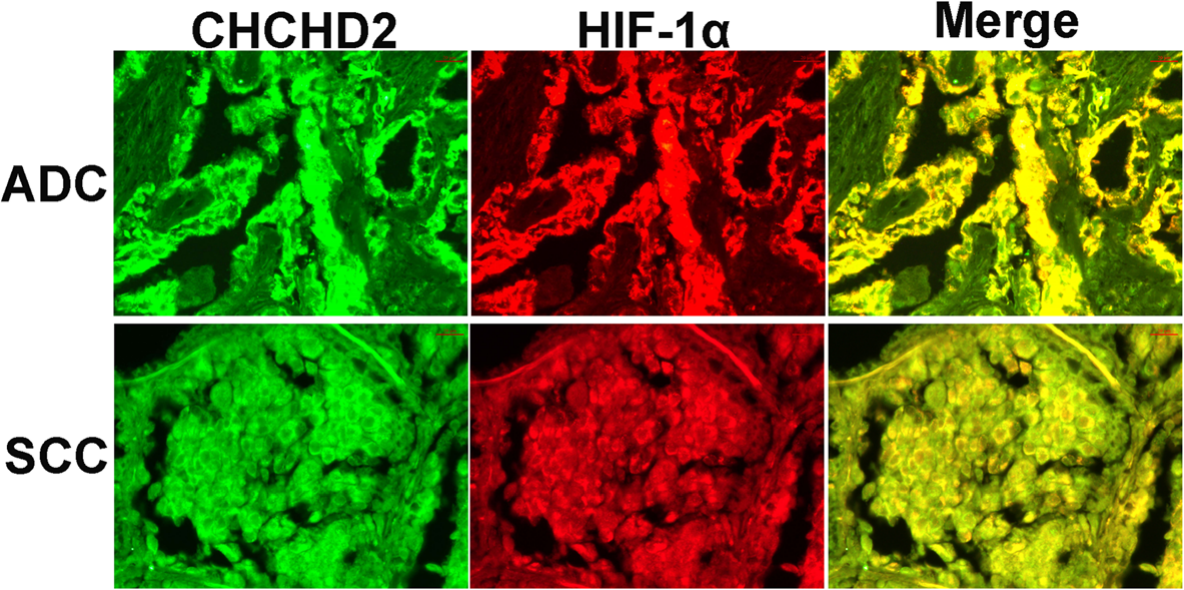
The co-localization of CHCHD2 and HIF-1α protein expressions were observed by Immunofluorescence. ADC: adenocarcinoma; SCC: squamous cell carcinoma (All images are magnified at × 400)

CHCHD2 and HIF-1α located in the cytoplasm and nucleus by immunohistochemisty staining. Furthermore, we performed an Immunohistofluorescence assay to investigate whether CHCHD2 and HIF-1α is co-localized. The results showed that co-localization phenomenon of CHCHD2 and HIF-1α in NSCLC existed both in ADC and SCC (Fig. 4).

In order to investigate the role of CHCHD2 in NSCLC and the correlation of CHCHD2 with HIF-1α expression, the correlation of CHCHD2 with HIF-1α expression and intensity according to immunohitochemical staining of these proteins were analyzed. There were anobvious correlation of CHCHD2 with HIF-1α expression in NSCLC, ADC, and SCC (P<0.001, r_s_>0.447). Similar correlations were displayed in differentiation (*P* < 0.05, r_s_>0.380) and lymph node metastasis (*P*<0.001, r_s_>0.494), Table 4.

**Table 4:** The correlation of CHCHD2 with HIF-1α protein expression in NSCLC

Group		CHCHD2	HIF-1α					
			–	+	++	+++	r_s_	*P*-value
NSCLC		–	7	2	2	0	0.526	0.000
		+	1	35	43	3		
		++	1	11	80	9		
		+++	0	0	5	10		
Histological type								
	Adenocarcinoma	–	1	0	1	0	0.447	0.000
		+	1	9	18	1		
		++	0	4	37	4		
		+++	0	0	3	4		
	Squamous caner	–	6	2	1	0	0.548	0.000
		+	0	26	25	2		
		++	1	8	43	5		
		+++	0	0	2	6		
Differentiation								
	High/ Moderate	–	5	2	1	0	0.550	0.000
		+	1	31	34	0		
		++	1	9	54	6		
		+++	0	0	2	6		
	Poor	–	2	0	1	0	0.380	0.004
		+	0	4	9	3		
		++	0	2	26	3		
		+++	0	0	3	4		
Lymph node metastasis								
	Lymph metastasis	–	2	1	1	0	0.515	0.000
		+	1	12	18	2		
		++	0	7	53	5		
		+++	0	0	5	9		
	Non-Lymph metastasis	–	5	1	1	0	0.494	0.000
		+	0	23	25	1		
		++	1	4	27	4		
		+++	0	0	0	1		

## Discussion

CHCHD2 is well-conserved among different species from humans to yeast, mouse and human CHCHD2 share 87% amino acid sequence identity [22]. Previous studies have found that CHCHD2 contains CHCH domain, while the proteins which contain the CHCH domain have diverse functions [23, 24]. Some studies have shown that the expression of CHCHD2 are related to glycolysis and translation [11, 12] or play an importent role in enhancing cell migration-promoting activity [12, 25]. However, the CHCHD2 expression on the prognosis of NSCLC has not been reported. The purpose of this study was to investigate the clinical significance of the expression of CHCHD2 in NSCLC and to reveal the possible mechanism of the expression CHCHD2 for the adverse prognosis of NSCLC.

Previously, human *CHCHD2* gene was determined overexpression in some of cancer tissues [1, 2]. In our study, we firstly provided evidence that CHCHD2 mRNA and protein overexpressed in NSCLC tissues by qRT-PCR and Western blot methods. Then based on the data of oncomine database, it was confirmed that the mRNA expression level of CHCHD2 in NSCLC was higher than that in normal tissue. Further more we detected the protein expression of CHCHD2 in 209 pairs of specimens by immunohistochemistry, the positive rate of CHCHD2 (94.7%) with NSCLC tissues was higher than that of normal tissues (11.0%) (*P* = 0.000). Consequently, both previous and our studies all showed that CHCHD2 overexpressed in cancerous tissues. These data suggest that CHCHD2 may be a new biomarker for lung cancer.

Subsequently, we found that the expression of CHCHD2 in NSCLC was notably associated with some clinical parameters of NSCLC, such as smoking (*P* = 0.045), tumor size (*P* = 0.000), differentiation degree (*P* = 0.034), TNM stage (*P* = 0.000), lymph metastasis (*P* = 0.000). These findings indicate that CHCHD2 may play an import role in the proliferation and metastasis of NSCLC. And in the report of Minchul Seo [10], overexpression of CHCHD2 can promote the migration of NIH3T3 fibroblasts cells, and knockdown of the endogenous CHCHD2 can reduce the motility of NIH3T3 fibroblasts cells. Combined with our results and existing reports, it is suggested that CHCHD2 may be related to the proliferation, invasion and metastasis of NSCLC.

In addition, we analyzed the relationship between tumor size, differentiation, TNM stage, lymph node metastasis and patient survival, these factors were all related to CHCHD2 expression. The results showed that patients with tumor size > 5 cm, poor differentiation, high TNM stage and lymph node metastasis had shorter median survival time and lower 5-year survival rates. These evidences indicated that tumor size, differentiation, TNM stage, lymph node metastasis would been important factors of surgical patients’ survival rates. Then we analyzed the relationship between the expression of CHCHD2 and HIF-1a and survival rates of patients. These results indicated that patients with high expression of CHCHD2 and HIF-1a had shorter median survival time and lower 5-year survival rates, which suggested that they were also important factors affected the survival rates of surgical patients. Therefore, CHCHD2 would been an adverse prognostic factor of NSCLC.

To further clarified CHCHD2 expression was a adverse prognostic factor of NSCLC, a Cox’s proportional hazards model was used for regression analysis. According to the results of univariate analysis, tumor size, grade of differentiation, TNM stage and lymph node metastasis showed significantly higher hazard ratios for adverse prognostic. Multivariate analysis showed that grade of differentiation, lymph node metastasis CHCHD2 and HIF-1α expressions were all showed significantly higher hazard ratios, which indicated that these factors would been independent prognostic factors of NSCLC. Thus, these evidences once again proved that CHCHD2 expression was a independent adverse prognostic factor of NSCLC. Due to the limitations of the sample size and follow-up, the median survival time in many groups could not be obtained. To compensate for these shortcomings, we intend to carry out further multicenter clinical studies, expand the sample size and enrich the means of detection [26].

Meanwhile, we found that HIF-1α was overexpression in NSCLC tissues and had a poor prognosis. Moreover, we found an interest problem that when CHCHD2 expressed in the nucleus, HIF-1α also expressed in the nucleus. It suggested that maybe CHCHD2 and HIF-1α are co-expressed in the nucleus of cells in NSCLC. Therefore we investigate the correlation of CHCHD2 with HIF-1α for the first. The correlation of CHCHD2 with HIF-1α was significant in regards to histological type, differentiation and lymph node metastasis (*P*<0.01, r_s_ >0.3). In order to further verify whether there was a co-localization phenomenon for the CHCHD2 and HIF-1α expression, immunofluorescence experiments were conducted. The results showed that the co-localization phenomenon existed. Thus, we suspect that if the expression of CHCHD2 accompanies the HIF-1α expression participated in the regulation signaling pathways, so that lung cancer cells could adapt to hypoxic environment and play a role in angiogenesis, invasion, metastasis and metabolism of lung cancer.

## Conclusions

This study showed that the expression of CHCHD2 and HIF-1α were higher in NSCLC tissues than normal tissues, and the expressions of these proteins were significantly associated with differentiation, TNM stage and lymphatic metastasis. Moreover, that high CHCHD2 and HIF-1α expression are associated with poor prognosis in NSCLC patients. All these signs indicated that CHCHD2 may play an important role in migration, invasion and metastasis of NSCLC. Present study indicate a potential role for CHCHD2 expression as an independent predictive factor of poor prognosis in NSCLC patients, and put forward the expressions of CHCHD2 maybe accompanies the HIF-1α expression, which will activate the hypoxic genes in this pathway. However, further study with larger sample size and cytological experiments are needed to confirm these results, and the specific pathway and mechanism driving this effect are also need to be further study.

## Data Availability

The datasets used and analyzed during the present study are available from the corresponding author on reasonable request.

## Abbreviations

ACS: American Cancer Society
ADC: Adenocarcinoma
CHCH: Coiled-coil-helix-coiled-coil-helix
CHCHD2: Coiled-coil-helix-coiled-coil-helix domain-containing protein 2
CI: Confidence interval
HIF-1: Hypoxia-inducible factor-1
MNRR1: Mitochondria Nuclear Retrograde Regulator 1
NSCLC: Lung cancer are non-small-cell lung cancer
OS: Overall survival
OxPhos: Oxidative phosphorylation
PMSF: Phenylmethanesulfonyl fluoride
pTNM: Pathological tumor/node/metastasis;
qRT- PCR: Quantitative real-time polymerase chain reaction
SCC: Squamous cell carcinoma
